# Mutations in the DNA processivity factor *POL30* predispose the *FLO11* locus to epigenetic instability in *S. cerevisiae*

**DOI:** 10.1242/jcs.262006

**Published:** 2024-12-17

**Authors:** Safia Mahabub Sauty, Ashley Fisher, Andrew Dolson, Krassimir Yankulov

**Affiliations:** Department of Molecular and Cellular Biology, University of Guelph, Guelph, Ontario N1G2W1, Canada

**Keywords:** PCNA, *POL30*, *TOF1*, *RRM3*, *FLO11*, Gene silencing, Replication fork barrier

## Abstract

The *FLO* genes in *Saccharomyces cerevisiae* are repressed by heterochromatin formation, involving histone deacetylases, transcription factors and non-coding RNAs. Here, we report that mutations in the processivity factor *POL30* (PCNA) that show transient derepression at the subtelomeres and the mating-type loci do not derepress *FLO* loci. However, deletions of the replisome stability factors *RRM3* and *TOF1* along with *pol30* mutations induced flocculation phenotypes. The phenotypes correlated with increased expression of reporter proteins driven by the *FLO11* promoter, the frequency of silent to active conversions of *FLO11*, and reduced expression of the regulatory long non-coding RNAs *ICR1* and *PWR1*. Alterations in the local replication landscape of *FLO11* indicate a link between defects in the fork protection complex and the stability of gene silencing. Analyses of these mutants at the subtelomeres and the *HMLα* locus showed a similar derepression phenotype and suggest transient instability of both active and silent states of *FLO11*. We conclude that *RRM3* and *TOF1* interact differentially with the *pol30* mutations to promote transient derepression or complete epigenetic conversions of *FLO11*. We suggest that the interaction between *POL30*, *RRM3* and *TOF1* is essential to maintain epigenetic stability at the studied loci.

## Teaser

**Summary:** The repression of a cell surface flocculin (Flo11p) in laboratory yeast strains is epigenetically maintained by the replication-coupled chromatin assembly pathway.

## INTRODUCTION

The genome of *Saccharomyces cerevisiae* harbors several regions that experience transcriptional silencing. The subtelomeres, the mating-type locus and the rRNA gene loci undergo position-dependent heterochromatic silencing mediated by the silent information regulator (SIR) family of proteins. In contrast, *FLO* gene silencing is independent of the *SIR* genes (Sauty et al., 2021; Verstrepen and Klis, 2006). *FLO* genes express cell surface proteins necessary for cell adhesion and flocculation in yeasts. In industrial and wild-type strains, flocculation is initiated as a response to stress or adverse conditions. Pathogenic yeast strains utilize this trait as a virulence factor (Verstrepen and Klis, 2006). However, most laboratory *S. cerevisiae* strains have been selected against flocculation and display a predominantly planktonic phenotype.

The *FLO* genes (*FLO1*, *FLO5*, *FLO9*, *FLO10* and *FLO11*) are positioned 20–40 kb away from the telomeres (Halme et al., 2004; Van Mulders et al., 2009). Previous studies have shown that *FLO11* expression alternates between ON and OFF states in certain strains (Bumgarner et al., 2012; Halme et al., 2004). This expression pattern is similar to the variegated expression observed at yeast subtelomeres (telomere position effect) or position effect variegation in *Drosophila* (Shaban et al., 2021; Yankulov, 2013). The *FLO11* locus is repressed by several histone deacetylases including Hda1p and Rpd3p. The stochastic active or silent expression of *FLO11* is also regulated by two long non-coding RNAs (lncRNAs), namely, *ICR1* and *PWR1* (Bumgarner et al., 2009; Winston, 2009). These are transcribed in opposite directions about 3000 bp away from the *FLO11* promoter. Competitive binding of two transcription factors, Slf1p (repressor) and Flo8p (activator), and the histone deacetylase complex Rpd3L toggles between the mutually exclusive transcription of *PWR1*, which promotes *FLO11* expression, and that of *ICR1*, which represses *FLO11* (Bumgarner et al., 2009). The specific mechanism and the rate of conversion between the active and silent states of expression are unknown.

The epigenetic silencing of *FLO11* is disrupted when it is moved to another genomic site with its original promoter, or by substituting the *FLO11* promoter with a different one in its native site (Halme et al., 2004). This implies a dual regulation mechanism, involving both promoter-specific and global regulatory influences on this gene. Recent studies have shown that the deletion of the genes *CAC1* (also known as *RLF2*) and *ASF1* and the helicase-encoding gene *RRM3* results in the derepression of *FLO* loci and increased flocculation (Rowlands et al., 2019; Shaban et al., 2023). *CAC1* and *ASF1* encode components of histone chaperones engaged in the replication-coupled disassembly and reassembly of nucleosomes (Rowlands et al., 2017). These observations suggest that the replication-coupled transmission of epigenetic marks is essential for the maintenance of silencing of *FLO* genes.

*RRM3* encodes a DNA helicase necessary to relieve the pausing of the forks at non-nucleosomal fork barriers (Ivessa et al., 2002; Makovets et al., 2004). There are ∼1400 natural replication pause sites in the genome of *S. cerevisiae* (Azvolinsky et al., 2006). The activity of *RRM3* is opposed by the fork protection complex (FPC), which is built by three proteins encoded by *TOF1*, *CSM3* and *MRC1*. Recent studies conducted in *Schizosaccharomyces pombe* and mammalian cells have shown that the Mrc1p (claspin) component of the FPC contains an H3/H4 histone-binding domain and acts as a chaperone during DNA replication (Charlton et al., 2024; Yu et al., 2024). In *S. cerevisiae*, a counterbalance between *TOF1–CSM3* and *RRM3* activity has been shown to regulate the termination of replication at the programmed replication fork arrest at the rRNA loci (Bastia et al., 2016; Mohanty et al., 2006). Both *TOF1* and *RRM3*, along with chromatin assembly factor 1 (CAF1) and *ASF1*, contribute to gene silencing at *SIR*-dependent silent loci (Rowlands et al., 2019; Shaban et al., 2023), but the interplay between these factors and the core DNA replication machinery remains enigmatic.

*POL30* (PCNA) encodes the homotrimeric sliding clamp at the core of the advancing replication fork (Moldovan et al., 2007). It is employed in the processing of Okazaki fragments, DNA repair, sister chromatid cohesion and the control of cell cycle (Moldovan et al., 2007). Three double amino acid substitution mutations, *pol30-6* (DD41,42AA), *pol30-8* (RD61,63AA) and *pol30-79* (LI126,128AA) have also pointed to a role of *POL30* in the coordination of DNA replication and chromatin reassembly. At the telomeres of *S. cerevisiae*, these mutations lead to a substantial loss in SIR protein-mediated gene silencing (Sauty and Yankulov, 2023; Zhang et al., 2000). At the mating-type loci, which utilize a similar mechanism of gene silencing, the same mutations only have a transient derepression effect and do not lead to a conversion to the active state (Brothers and Rine, 2019). The effects of these mutations at the SIR-independent *FLO11* locus have not been addressed. Detailed investigations of the three *pol30* alleles have shown that more than one *POL30*-mediated pathway is used in chromatin maintenance. At least one of them involves the direct interaction of Pol30p with CAF1, a histone chaperone engaged in the assembly of H3/H4 histone tetramers on newly synthesized DNA (Jeffery et al., 2013; Krawitz et al., 2002; Sharp et al., 2001; Yang et al., 2008; Zhang et al., 2000). It has been shown that the three *pol30* mutants poorly interact with the Cac1p subunit of CAF1 (Sauty and Yankulov, 2023; Zhang et al., 2000) and that Cac1p and Rrm3p compete for the interaction with Pol30p (Wyse et al., 2016); however, the links between *POL30* and other histone chaperones or other factors that affect gene silencing have not been addressed in detail.

We recently demonstrated that the deletions of *RRM3* and *TOF1* lead to mild loss-of-silencing defects, which are exacerbated by the destruction of histone chaperones (Rowlands et al., 2019; Shaban et al., 2023; Sauty and Yankulov, 2023). These two genes also play major roles during the pausing of replication forks (Bastia et al., 2016; Shyian et al., 2020). However, it remains unclear whether the pausing of replication forks is linked to the maintenance of chromatin. In this study, we first asked whether *pol30-6*, *pol30-8* and *pol30-79* disrupt the silencing of the *FLO11* locus. We then used the same alleles to sensitize gene silencing and test the effects of the deletion of *RRM3* and *TOF1* as well as the pausing of forks on gene expression at *FLO11* and other silenced loci.

## RESULTS

### Flocculation phenotypes in *pol30* mutants

**Fig. 1.**
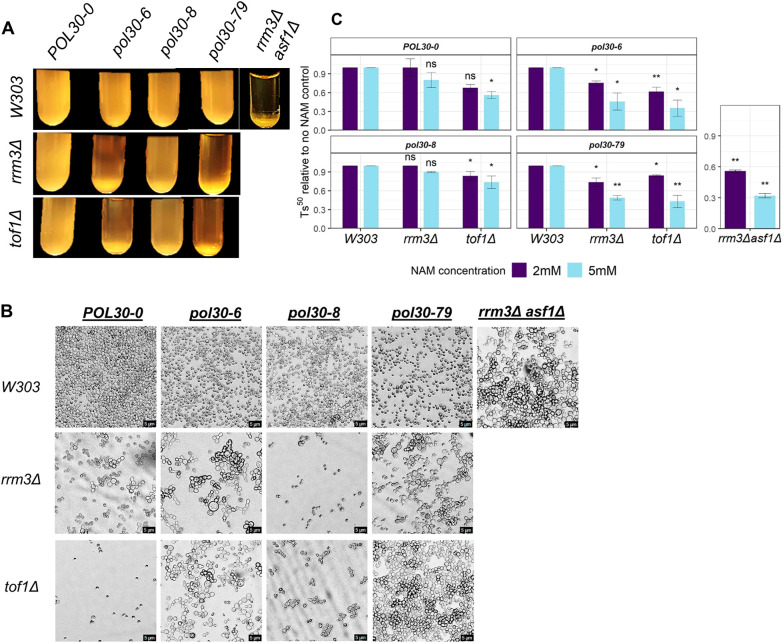
**Flocculation phenotype in *pol30* mutant strains.** (A) Cells were grown in liquid culture on a spinning wheel and allowed to rest for 15 min before images were taken. (B) The indicated strains were grown without shaking for 24 h and imaged with a Leica Stellaris confocal microscope equipped with a 40× objective using the brightfield channel. Scale bars: 5 μm. (C) Quantification of sedimentation rates of *pol30* mutants induced with nicotinamide (NAM). Cells were grown overnight in the presence of 0, 2 and 5 mM NAM. Sedimentation rates were calculated as the time required to achieve 50% clearance of the culture volume (Ts^50^) and expressed as a factor of Ts^50^ of untreated culture. The bars on the *y*-axis represent the average of three independent experiments, and the error bars represent standard deviation. Asterisks represent statistical significance compared to the wild-type *W303* strain for each allele and concentration. Significance was determined by one-way ANOVA and post hoc Dunnett's test. ns, not significant; *P<0.05; **P<0.01.

The yeast laboratory strains *W303* and *BY4743* and their derivatives do not flocculate under normal conditions. In earlier studies, we have shown that combinations of deletions of histone chaperones, histone deacetylases and the replicative helicase *RRM3* lead to the derepression of *FLO* genes and to apparent flocculation phenotypes (Rowlands et al., 2019; Shaban et al., 2023). To test whether the loss of silencing of *FLO* loci is replication dependent, we asked whether *POL30* mutations (*pol30-6*, *pol30-8* and *pol30-79*) would have a similar effect on the flocculation of these strains. These very same mutations have shown loss of silencing at the subtelomeres and mating-type loci (Brothers and Rine, 2019; Sauty and Yankulov, 2023; Zhang et al., 2000). We grew these strains in liquid cultures and assessed the sedimentation rates as in Rowlands et al. (2019). No apparent flocculation was observed in any of the *pol30* mutants (Fig. 1A). However, the deletions of *RRM3* or *TOF1* in these strains increased the sedimentation rates in the *pol30-6* and *pol30-79* strains, but not in the *pol30-8* strain (Fig. 1A). The observed flocculation was less pronounced compared to the flocculation of the control strain *rrm3Δ asf1Δ* (Rowlands et al., 2019).

In agreement with the sedimentation analyses, microscopy inspection of the cultures revealed small flocs in *pol30-6 rrm3Δ*, *pol30-79 rrm3Δ*, *pol30-6 tof1Δ* and *pol30-79 tof1Δ* (Fig. 1B). However, these flocs were substantially smaller than the ones observed in *rrm3Δ asf1Δ.*

We then tested sedimentation rates in the presence of nicotinamide (NAM). NAM inhibits NAD^+^-dependent histone deacetylases and has been shown to expedite floc formation (Rowlands et al., 2019). As expected, NAM had no effect in the single *pol30* mutants (Fig. 1C). However, deletion of both *RRM3* and *TOF1* increased the sedimentation rates with increasing NAM concentration in the *pol30-6* and *pol30-79* strains, but not in the wild-type *POL30-0* and *pol30-8* strains. Thus, we concluded that the silencing of *FLO* loci is moderately compromised in *pol30-6* and *pol30-79* mutants and is exacerbated the by the deletions of *TOF1* and *RRM3*, leading to a mild flocculation phenotype.

### Analyses of FLO11–yEGFP expression

To further investigate the link between DNA replication and the loss of silencing at *FLO* loci, we replaced the *FLO11* open reading frame (ORF) with a *yEGFP* reporter driven by the native *FLO11* promoter and analyzed its expression as in Rowlands et al. (2019).

**Fig. 2.**
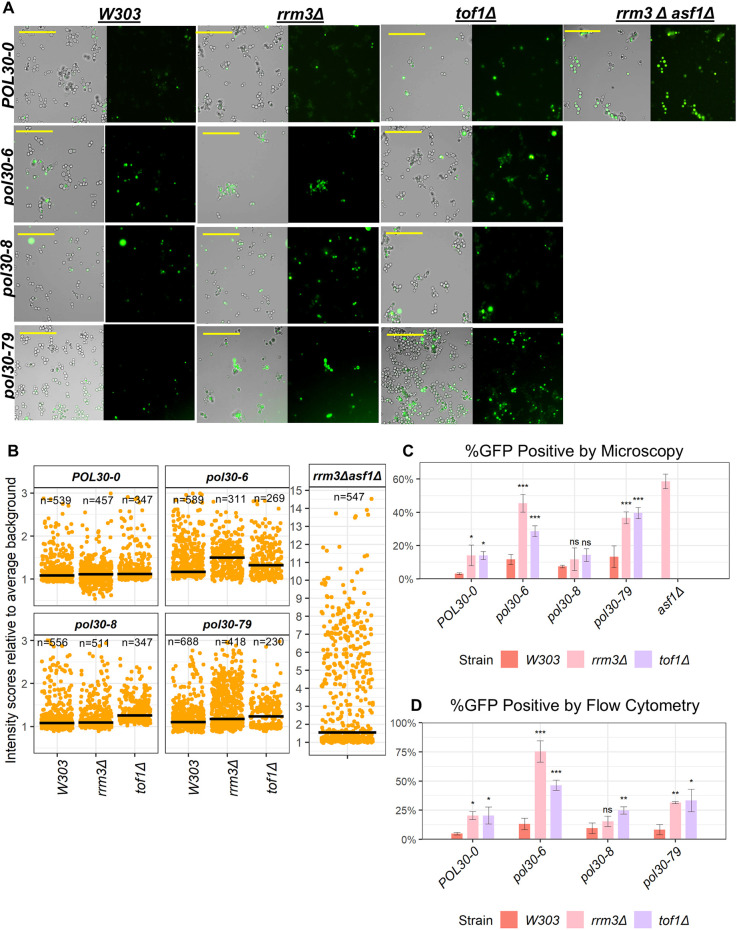
**Analysis of the expression of *FLO11-yEGFP*.** (A) Cell suspension images of cells harboring *FLO11-yEGFP*. The strains harboring *FLO11-yEGFP* in *pol30* mutants (shown on the left) combined with the deletions of *RRM3* and *TOF1* (shown on the top) were grown overnight in liquid cultures and vortexed vigorously to disperse flocs before imaging with brightfield and green fluorescence channels using a Leica DM600B microscope. Scale bars: 20 μm. (B) Scatter plots showing the GFP intensity scores of individual cells relative to the average background intensity imaged in three independent experiments. The black bars indicate median values. *n* represents the number of cells plotted. (C) The percentages of GFP-positive and -negative populations in the microscopy images were calculated by setting an upper threshold of 160% above the average background intensity deemed as GFP negative. Data from three independent experiments were averaged to create a bar plot. Error bars indicate standard deviation. Asterisks represent statistical significance compared to the wild-type *POL30-0* within each strain. Significance was determined by one-way ANOVA and post hoc Dunnett's test. ns, not significant; *P<0.05; **P<0.01; ***P<0.001. (D) Cell suspensions of the indicated strains were vigorously vortexed and analyzed by flow cytometry with a green fluorescence filter. Representative images of the flow cytometry plots are shown in Fig. S1. Data from at least three independent experiments were averaged to create a bar plot. Error bars indicate standard deviation. Asterisks represent statistical significance compared to the wild-type *W303* within each strain. Significance was determined by one-way ANOVA and post hoc Dunnett's test. ns, not significant; *P<0.05; **P<0.01; ***P<0.001.

First, we measured the levels of yEGFP expression in individual cells. The strains were grown in liquid cultures, spread on microscopic slides and inspected by fluorescence microscopy (Fig. 2A). The intensity of yEGFP signals in at least 200 cells from three independent experiments was measured. The intensities of 50 cell-free regions in each image were deemed background intensity. The yEGFP signal in the cells were normalized by the background signal and plotted in a scatter graph (Fig. 2B). These analyses demonstrated elevated levels of median yEGFP signal distribution in the mutants with mild flocculation phenotype (*pol30-6 rrm3Δ*, *pol30-79 rrm3Δ*, *pol30-6 tof1Δ*, *Δpol30-8 tof1* and *pol30-79 tof1Δ*). The overall average signals in the analyzed mutants ranged from 1.25 to 3 times above the respective background signals and were substantially lower compared to the signals in the control *rrm3Δ asf1Δ* strain, which ranged from 1.5 to 15 times above the background.

We then calculated the proportions of cells expressing yEGFP by setting a GFP-positive threshold at 160% above the background signal. It has been previously shown that this threshold can detect cells with GFP-positive or GFP-negative cells with more than 90% accuracy (Shaban et al., 2023). These analyses revealed about 3% yEGFP-positive cells in the *POL30-0* strain (Fig. 2C). This percentage increased 4-fold in the *rrm3Δ* and *tof1Δ* genetic backgrounds. The deletion of *RRM3* increased the yEGFP-positive population by 4-fold in *pol30-6* and 3-fold in *pol30-79*. The deletion of *TOF1* produced an approximately 2-fold increase in the yEGFP-positive population in *pol30-6* and a 3-fold increase in *pol30-79*. The *pol30-8* strain did not show a significant additive effect of the percentage of yEGFP-positive population upon deletion of either *RRM3* or *TOF1* (Fig. 2C). The percentage of yEGFP-positive cells in the control *rrm3Δ asf1Δ* strain was slightly higher than the percentage in the mildly flocculating strains (Fig. 2C). We concluded that the difference in the magnitude of flocculation between the control *rrm3Δ asf1Δ* and the *pol30* mutant strains was due to the levels of expression of *FLO11* rather than the proportion of cells expressing *FLO11*.

We also attempted to estimate the percentage of yEGFP-positive cells by flow cytometry. Representative images of the flow cytometry plots are shown in Fig. S1. In agreement with the microscopic analyses (Fig. 2B,C), we observed a continuous distribution of yEGFP signals in the individual cells rather than distinct populations of yEGFP^+^ and yEGFP^−^ cells (Fig. S1). None of the *pol30* mutations produced a substantial upward shift of yEGFP^+^ cells. Upon deletion of *RRM3* and *TOF1*, there was a 4-fold increase in the population of cells with signals above the yEGFP-negative threshold in *POL30-0* and *pol30-79* (Fig. 2D). *pol30-6* showed a 5-fold increase in the *rrm3Δ* background and a 3-fold increase in the *tof1Δ* background. *pol30-8* did not show any additive effect upon *RRM3* deletion but a modest 2-fold increase in the *tof1Δ* background (Fig. 2D).

In summary, both *RRM3* and *TOF1* interact with the *pol30-6* and *pol30-79* mutations to maintain the silencing at the *FLO11* locus. Loss of these interactions causes mild flocculation and/or epigenetic conversion from the silent to an active state of *FLO11*.

### Epigenetic conversions of *FLO11*

**Fig. 3.**
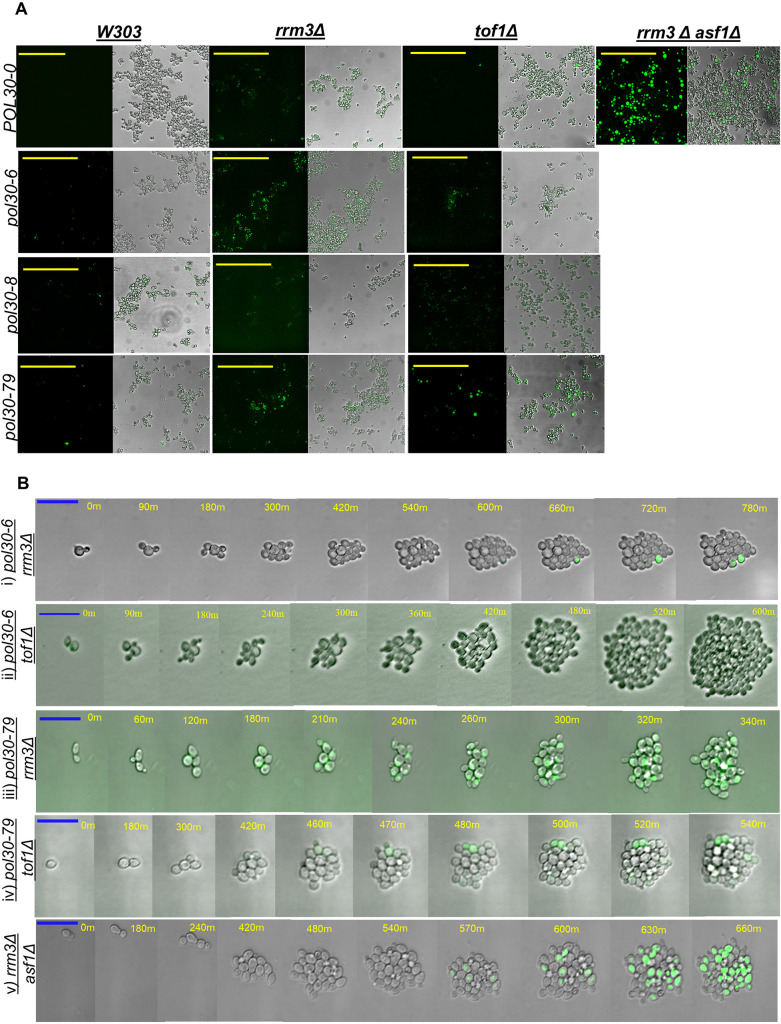
**Analysis of frequency of epigenetic conversions of *FLO11-yEGFP*.** (A) Cells harboring *FLO11-yEGFP* were serially diluted in 96-well plates and grown overnight without shaking. Cell clusters were imaged with brightfield and green fluorescence channels using a Leica DMi8 microscope. Scale bars: 100 μm. (B) Terminally diluted cells were grown under agar slabs and imaged for 14 h using brightfield and green fluorescence channels. Images of the indicated strains are shown at comparable growth stages. The brightfield to GFP signal ratio was adjusted to better capture the ‘active’ state of the gene in strains with low yEGFP intensity. Timestamps are shown in minutes. Scale bars: 20 μm. The calculated frequency of epigenetic conversions is shown in Table 1. Images represent three independent independent experiments.

The analyses in Fig. 2 demonstrated lower levels of flocculation and the expression of the FLO11–yEGFP reporter compared to those in *rrm3Δ asf1Δ* cells. These levels could be a consequence of lower levels of *FLO11* expression in cells that have lost silencing, or of infrequent active-to-silent and silent-to-active conversions of the gene. To address these possibilities, we terminally diluted cultures in 96-well plates, grew them without shaking for 12 h, and inspected wells that initially contained one or two cells. This approach revealed the formation of clusters of yEGFP-positive cells in the *pol30-6 rrm3Δ*, *pol30-79 rrm3Δ*, *pol30-6 tof1Δ* and *pol30-79 tof1Δ* mutants, but not in the *POL30-0* and *pol30-8* strains (Fig. 3A). The control *rrm3Δ asf1Δ* strain showed flocs of heterogenous size and yEGFP intensity. The results correlated with the flocculation phenotypes and the percentage of yEGFP-positive cells as measured in Fig. 2 and suggested that silent-to-active conversion contributes to these phenotypes. Because flow cytometry could not identify distinct populations of yEGFP^+^ and yEGFP^−^ cells, we measured the frequency of such conversions by time-lapse fluorescence microscopy.

**Table 1.**
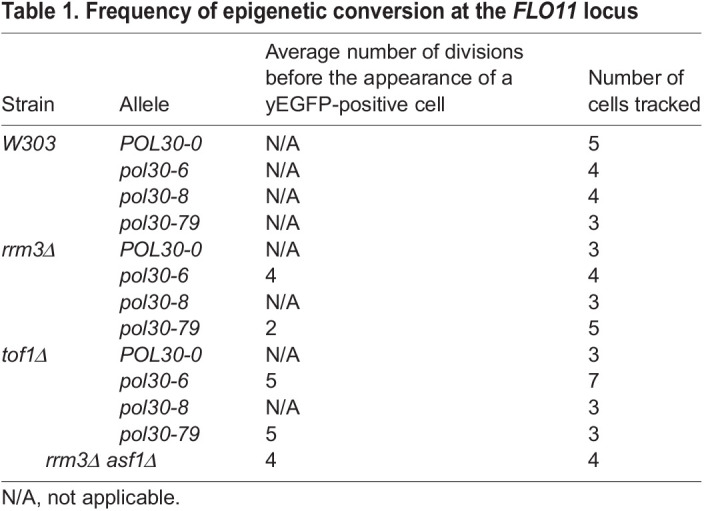
Frequency of epigenetic conversion at the *FLO11* locus

We spread diluted cultures on microscopic slides, covered them with a slab of agar and tracked the divisions of yEGFP-negative cells for 14 h (Movie 1, Fig. 3B). The frequency of epigenetic conversion was recorded as the number of cell divisions before the first appearance of a yEGFP-positive cell with signals higher than 160% above the background. At least three single cells per strain were tracked (Table 1). Consistent with the microscopy distribution data, the analyses showed that *pol30-6 rrm3Δ* and *pol30-79 tof1Δ* displayed bimodal yEGFP*^+^* and yEGFP*^−^* expression with a distinct appearance of a yEGFP-positive cell (Fig. 3B, panels i and iv). The frequency of these epigenetic conversions was comparable to that in the control strain *rrm3Δ asf1Δ* (Table 1). Consistent with the data in Fig. 2, the *rrm3Δ asf1Δ* strain showed a higher number of high-intensity yEGFP-positive cells (Fig. 3B, panel v). *pol30-79 rrm3Δ* showed yEGFP expression as early as the second division (Fig. 3B, panel iii). However, the yEGFP intensity was low across all subsequent cell divisions compared to that in the positive control strain. Similar events were recorded for the *pol30-6 tof1Δ* strain (Fig. 3B, panel ii)*.* All *pol30-6 tof1Δ* cells showed a green glow that either disappeared or converted to an yEGFP-positive state during the course of the experiment. The intensity of these GFP-positive cells was very low compared to that in the control strain *rrm3Δ asf1Δ*, and the overall GFP signal was digitally enhanced to detect the different states of yEGFP expression in this strain. The *pol30* mutants that did not show a flocculation phenotype or increased yEGFP signals in Fig. 3A did not produce any detectable yEGFP signal in the time-lapse experiments either. We reported the conversion frequency of these strains as ‘not applicable’ (N/A) while being cognizant of the possibility that a conversion could happen during a cell division beyond our experimental timeframe (Table 1).

We concluded that the mild flocculation phenotypes in *pol30-6 rrm3Δ* and *pol30-79 tof1Δ* are caused by epigenetic silent-to-active conversions. *pol30-79 rrm3Δ* and *pol30-6 tof1Δ* showed the possibility of transient activation of the *FLO11* gene. The possibility of a transient derepression effect in these mutants needed to be further investigated.

### Transient derepression of genes at the *HMLα* locus and the *VIIL* subtelomere

We have previously shown that the deletion of *RRM3* in conjunction with *pol30-79* leads to transient derepression of reporters at the *VIIL* telomere (Sauty and Yankulov, 2023). Others have shown transient loss of silencing at the *HMLα* locus (Brothers and Rine, 2019). Because we lacked a reliable assay for the detection of transient derepression of *FLO11*, we analyzed these two loci to address the possibility that mutations in *TOF1* and *POL30* lead to transient derepression.

**Fig. 4.**
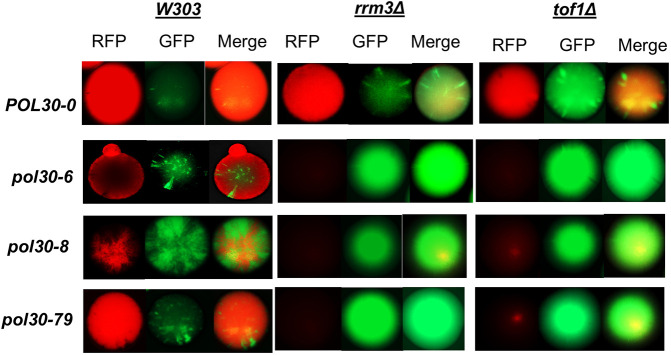
**Analysis of gene silencing at the *HMLα* locus.** Strains harboring a CRASH construct at the *HMLα* locus were grown in the presence of hygromycin B to select for the RFP-positive state. Cells were then spread on non-selective medium and grown for 2–5 days before imaging single colonies. Images represent three independent experiments.

The irreversible Cre-reported altered states of heterochromatin (CRASH) assay detects transient loss of silencing at the *HMLα* locus (Brothers and Rine, 2019). The events of transient loss of silencing are recorded as a conversion from *RFP* to *yEGFP* expression from a reporter cassette and the appearance of green sectors in a red colony (Brothers and Rine, 2019). All *pol30* mutants harboring the CRASH cassette were grown overnight in liquid media with 200 μg/ml hygromycin to select for the *RFP*-expressing state. The cultures were then serially diluted, spotted on non-selective media and incubated at 30°C for 3–5 days. As previously reported, all *pol30* mutations showed green sectoring, with *pol30-8* showing the highest instability of this locus (Brothers and Rine, 2019) (Fig. 4). The deletion of *RRM3* or *TOF1* increased the green sectoring in *POL30-0*, pointing to a similar loss of repression by these gene deletions (Fig. 4). However, in the *pol30* mutant background, these deletions produced green-only colonies (Fig. 4). We concluded that the deletion of *RRM3* and *TOF1* creates a high level of instability in the mating-type loci of the *pol30* mutants and cannot be studied by this assay. For this reason, we addressed this question with a dual reporter at the *VIIL* telomere as in Sauty and Yankulov (2023) and Shaban et al. (2023).

**Fig. 5.**
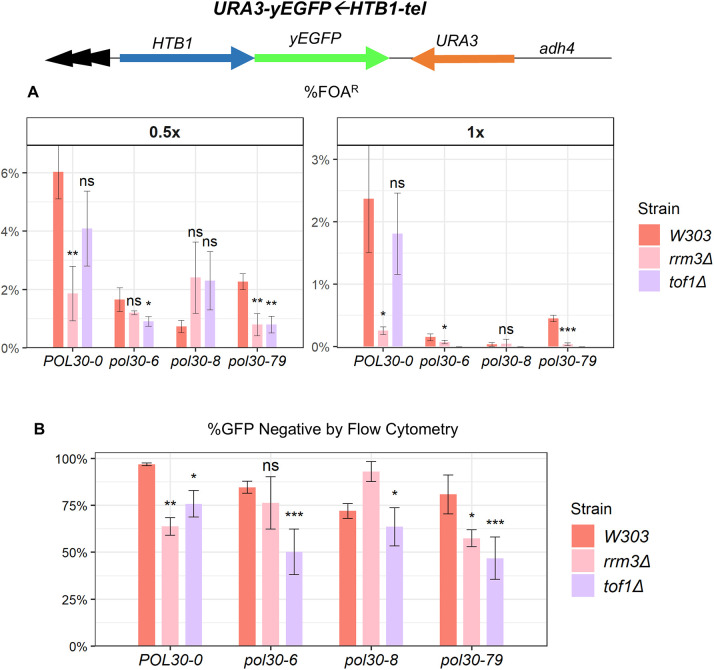
**Analysis of gene silencing at the *VIIL* subtelomere*.*** (A) Strains harboring the *adh4-URA3-yEGFP←HTB1-tel* reporter at the *VIIL* locus were analyzed using the 5-FOA assay. The percentage of cells with silenced *URA3* (%FOA^R^) at 0.5× and 1×5-FOA concentrations was recorded. The bar graph represents the average of three independent experiments. Error bars indicate standard deviation. Asterisks represent statistical significance compared to the wild-type *W303* within each strain and 5-FOA concentration. (B) yEGFP expression at the *VIIL* locus was analyzed using flow cytometry. Representative images of the flow cytometry plots are shown in Fig. S2. The average percentage of the GFP-negative population calculated from three independent experiments was plotted. Error bars indicate standard deviation. Asterisks represent statistical significance compared to the wild-type *W303* within each strain. Significance was determined by one-way ANOVA and post hoc Dunnett's test. ns, not significant; *P<0.05; **P<0.01; ***P<0.001.

We inserted a *URA3-yEGFP←HTB1-tel* construct at the *VIIL* locus in the *pol30* mutant strains with *TOF1* deletion. We used the highly sensitive 5-fluoroorotic acid (5-FOA) assay to measure the expression of *URA3* in the strains at 0.5× and 1× concentration of 5-FOA (Fig. 5A). Loss of silencing was recorded as the percentage of 5-FOA-resistant cells (%FOA^R^) cells as in Sauty and Yankulov (2023). As previously observed, deletion of *RRM3* showed an additive effect on the loss of silencing of *URA3* at both concentrations of 5-FOA in the *POL30-0* and *pol30-79* strains. However, the percentage of the 5-FOA-resistant population recorded was different at each concentration of 5-FOA, suggesting gradient *URA3* expression instead of outright silent-to-active conversion (Sauty and Yankulov, 2023) (Fig. 5A). Following the same trend, deletion of *TOF1* in *pol30* mutants showed an additive effect at different 5-FOA concentrations. At 0.5× concentration, deletion of *TOF1* produced a 4% 5-FOA-resistant population, which was further lowered to a <1% 5-FOA-resistant population in the *pol30-6* and *pol30-79* strains. The *pol30-8* strain did not show an additive effect with the deletion of these two genes (Fig. 5A). At 1× concentration, all *pol30* mutants showed an exacerbated loss of *URA3* silencing, causing the majority of cells to lose viability on 5-FOA medium.

We followed up by analysis of yEGFP expression from the same reporter using flow cytometry and plotted the percentage of the yEGFP-negative population (Fig. 5B). Representative images of the flow cytometry plots are shown in Fig. S2. The yEGFP-negative population is the functional equivalent of the 5-FOA-resistant population and is shown along with the previously published data of wild-type and *rrm3Δ* strains in Sauty and Yankulov (2023). We observed an upwards shift of yEGFP signals in *pol30* mutants with *TOF1* deletion, but not distinct populations of yEGFP*^+^* and yEGFP*^−^* cells (Fig. S2). The cells with signals above background followed the trend of yEGFP expressed from the *FLO11* promoter but did not reach the very high proportion of 5-FOA-sensitive cells. Combined, the results in Figs 4 and 5 (CRASH assays and *URA3-yEGFP←HTB1-tel* c assays) strongly suggest that the deletion of *TOF1*, similar to the deletion of *RRM3*, leads to a transient derepression of the silent state of the genes at the *HMLα* locus and the *VIIL* telomere.

### Effects of altering the local replication dynamics at *FLO11*

*POL30*, *TOF1* and *RRM3* encode proteins acting at the advancing replication fork (Rowlands et al., 2017; Shyian et al., 2020). Additionally, *TOF1* and *RRM3* are known to regulate the pausing of forks at hundreds of sites within the budding yeast genome (Azvolinsky et al., 2006; Ivessa et al., 2000). Given this, our analyses suggest that the passage of imperfect replication forks or its pausing, or both, could lead to the loss of silencing at *FLO11*. We addressed these possibilities by disrupting the local dynamics of DNA replication in the vicinity of this locus.

**Fig. 6.**
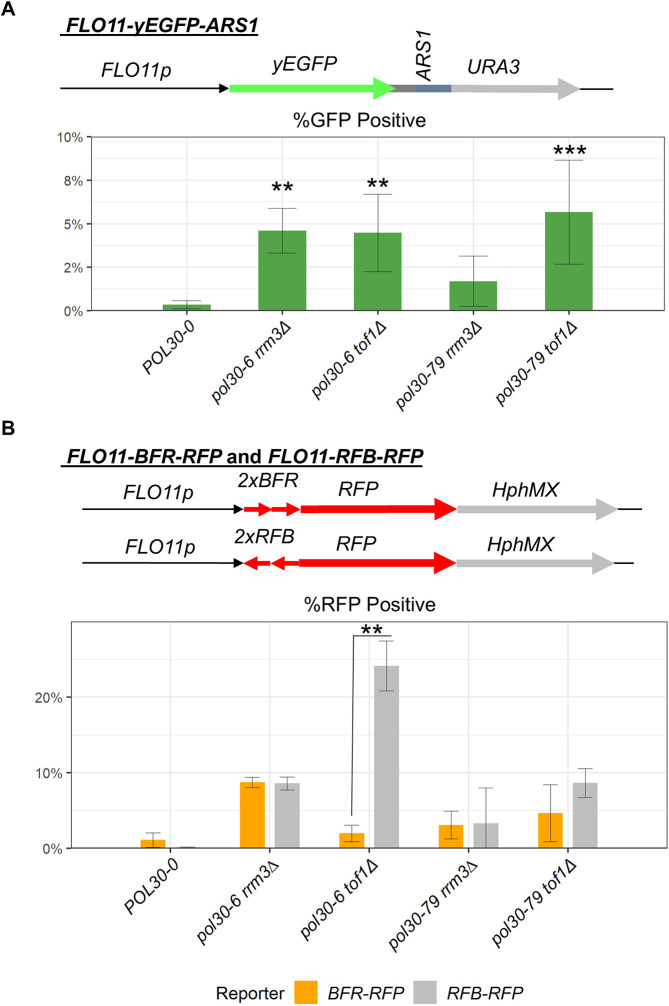
**Analysis of the expression of the *FLO11-yEGFP-ARS1*, *FLO11-BFR-RFP* and *FLO11-RFB-RFP* reporter constructs.** (A) The *FLO11-yEGFP-ARS1* construct (shown on top) was inserted in the *FLO11* locus of the strains listed on the horizontal axis and yEGFP expression was analysed using flow cytometry. Representative images of the flow cytometry plots are shown in Fig. S3A. The percentages of GFP-positive populations from at least three independent experiments were plotted. Error bars indicate standard deviation. Asterisks represent statistical significance between two fragments within each strain. Significance was determined by two-tailed unpaired Student's *t*-test. **P<0.01; ***P<0.001. (B) The *FLO11-BFR-RFP* and *FLO11-RFB-RFP* constructs, respectively, were inserted in the *FLO11* locus of the strains listed on the horizontal axis and RFP expression was analysed using flow cytometry. Representative images of the flow cytometry plots are shown in Fig. S3B,C. The percentages of RFP-positive populations from at least two independent experiments were plotted. Error bars indicate standard deviation. Asterisks represent statistical significance compared to *POL30-0* within each fragment. Significance was determined by one-way ANOVA and post hoc Dunnett's test. **P<0.01.

*FLO11* is positioned in the middle of a replicon and is replicated co-directionally with transcription (Kara et al., 2021; Liu et al., 2023; Lo and Dranginis, 1996). To alter the normal patterns of DNA replication, we inserted the strong *ARS1* origin downstream of the *FLO11-yEGFP* reporter in the control *POL30-0* strain and in the four strains (*pol30-6 tof1Δ*, *pol30-6 rrm3Δ*, *pol30-79 tof1Δ* and *pol30-79 rrm3Δ*) that have already shown derepression of *FLO11*. We then analyzed by flow cytometry the expression of yEGFP in individual cells (Fig. 6A). Representative images of the flow cytometry plots are shown in Fig. S3A. Again, we did not detect two distinct populations of yEGFP^+^ and yEGFP^−^ cells, but a gradient of cells with increasing GFP signals (Fig. S3A). These observations reiterate the possibility of frequent active-to-silent and silent-to-active conversions or transient derepression of the reporters at the *FLO11* locus. In addition, the overall signals in the *FLO11-yEGFP-ARS1-*harboring strains were lower compared to those in the *FLO11-yEGFP*-harboring strains. Although we do not entirely understand the reason for this decline in yEGFP expression, we attribute the differences to the presence of *ARS1*, which might interfere with the transcription of the *yEGFP* ORF or act as a proto-silencer at this location.

The analyses of the flow cytometry data demonstrated that the presence of *ARS1* did not lead to significant changes in the patterns of expression of yEGFP in the analysed mutants. Similarly to the case of the *FLO11-yEGFP* reporter (Fig. 2), in the *pol30-6 tof1Δ*, *pol30-6 rrm3Δ* and *pol30-79 tof1Δ* strains, the expression of yEGFP increased up to 4-fold relative to that in the *POL30-0* strain (Fig. 6A). In the *pol30-79 rrm3Δ* strain, the increase was about 2-fold (Fig. 6A). The patterns of expression suggest that if the firing of *ARS1* causes collisions with the transcription of *FLO11-yEGFP*, such collisions do not lead to increased derepression of the *FLO11* locus upon the deletion of *RRM3* or *TOF1* in conjunction with the *pol30-6* mutation. In the wild-type *POL30-0* strain, such collisions would have no detectable consequences. However, further analyses in other mutants are needed to conclusively address the effects of a nearby origin on the expression of silenced genes.

In a separate set of experiments, we engineered two reporters containing the *FLO11* promoter followed by two directional replication fork barriers (RFB) sites and the red fluorescent protein (*RFP*) ORF (Fig. S4). The two RFB sites were derived from the fork barrier in the rRNA loci (Castán et al., 2017) and were inserted in either the direction of transcription and replication (labeled as *BFR-RFP*, allowing replication through the barrier) or in the opposite direction (labeled as *RFB-RFP*, capable of pausing the replication fork) (Fig. S4, Fig. 6B). RFB sites are known to bind Fob1p (Castán et al., 2017) and can potentially interfere with the activity of the *FLO11* promoter. For this reason, we inserted the two RFB sites 60 bases downstream of the ATG codon and in-frame with the *RFP* ORF, thus producing an RFB–RFP fusion protein. Under these circumstances, the first RFB site is separated from the TATA box of *FLO11* by 110 bp.

We introduced these constructs in the control *POL30-0* strain and in the four mutants analyzed in Fig. 6A and determined the expression of RFB–RFP using flow cytometry. Representative images of the flow cytometry plots are shown in Fig. S3B. We observed weak RFP^+^ signals, possibly because of the 2×*RFB* tag, but were still able to detect differences between the strains harboring *RFB-RFP* and *BFR-RFP* (Fig. 6B).

In the control *POL30-0* strain, the presence of *RFB* in either orientation did not lead to detectable RFP× signals (Fig. 6B). These results suggested that pausing of the fork alone cannot dramatically alter the silencing of the *FLO11* locus. Next, we compared the expression of *BFR-RFP* and *RFB-RFP* in each of the mutant strains. Although the percentages of RFP-positive cells in all mutants were higher than that in the *POL30-0* strain, the orientation of the two RFBs did not make significant differences in the expression of *RFP* in the *pol30-6 rrm3Δ*, *pol30-79 tof1Δ* and *pol30-79 rrm3Δ* strains. However, in the *pol30-6 tof1Δ* strain, the presence of RFB produced a significant 8-fold increase in the percentage of RFP-positive cells. It is therefore conceivable that the pausing of a defective fork and, in particular, defect in the function of the FPC, would lead to derepression of this locus.

### Transcription of the lncRNAs *ICR1* and *PWR1*

**Fig. 7.**
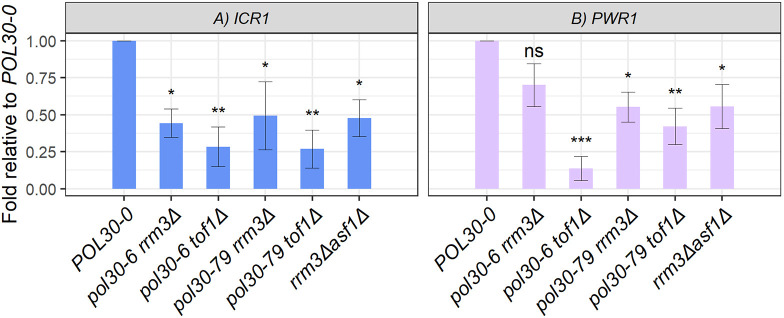
**Analysis of long non-coding RNA expression.** Total RNA was extracted from the indicated strains and the abundance of the long non-coding RNAs (lncRNAs) *ICR1* (A) and *PWR1* (B) was measured in triplicate by RT-qPCR. For each sample the signals of the lncRNAs were normalized to *ACT1*, then plotted against the normalized value in *POL30-0* strain. Average numbers for two independent biological replicates are shown. The outcome in each biological replicate is shown in Fig. S5. Error bars indicate standard deviation. Asterisks represent statistical significance compared to *POL30-0*. Significance was determined by one-way ANOVA and post hoc Dunnett's test. ns, not significant; *P<0.05; **P<0.01; ***P<0.001.

*FLO11* has one of the longest and most complex promotors in the genomes of budding yeasts. It is regulated by multiple positive and negative factors, including the lncRNA *ICR1*, the transcription of which is necessary for triggering the repression of *FLO11* (Bumgarner et al., 2009; Winston, 2009). The transcription of *ICR1* in turn is regulated by the transcription of another lncRNA, *PWR1*, over the promotor of *ICR1* (Bumgarner et al., 2009; Winston, 2009). We tested whether the transcription of these two lncRNAs in the mutants could be correlated to the loss of silencing of *FLO11*. RNA was isolated from the *POL30-0* and the mildly flocculating strains and subjected to real-time quantitative PCR (RT-qPCR) with primers specific for the *ICR1* and *PWR1* transcripts. We detected the presence of both *ICR1* and *PWR1* transcripts in the *POL30-0* strain (Fig. 7A,B). In two independent biological replicates, we observed a 2- to 4-fold decrease in the abundance of the *ICR1* transcript in the flocculating mutant strains (Fig. 7A). This outcome is consistent with a previous report, which indicated that the transcription of *ICR1* is necessary for the silencing of *FLO11* (Bumgarner et al., 2009). The abundance of *PWR1* decreased about 2-fold in the mildly flocculating mutants and up to 8-fold in the *pol30-6 tof1Δ* mutant (Fig. 7B). However, this substantial difference was not reflected in the abundance of the *ICR1* transcript, the expression of *FLO11-yEGFP* or the phenotype of the strain. We do not understand the reason for this reduction in the abundance of *PWR1*.

These experiments demonstrated that the loss of silencing of *FLO11* is accompanied by the reduced transcription of both *ICR1* and *PWR1.* At present, we cannot determine whether the transcription of *PWR1* or the passage of the imperfect replication fork, or both, are responsible for the reduced transcription of *ICR1*.

## DISCUSSION

### *FLO11* silencing is directly connected to the passage of replication forks

The silencing at *FLO* loci is maintained by heterochromatin formation involving class I and II histone deactetylases, transcription factors and lncRNAs. Previous studies have shown that the deletion of the histone chaperones *CAC1*, *ASF1*, *HIR1*, the histone deacetylase *HDA1*, in combination with the deletion of the helicase *RRM3* and FPC subunit *TOF1*, reconstitute the flocculation phenotype in laboratory yeast strains and show increased *FLO11* expression (Rowlands et al., 2019; Shaban et al., 2023).

Here, we asked whether the maintenance of heterochromatin-mediated gene silencing at *FLO* loci is directly connected to the core replication machinery. To address this question, we analyzed *POL30* mutations in conjunction with *RRM3* and *TOF1*. We showed epistatic interactions of *RRM3* and *TOF1* with mutant *pol30* alleles that led to mild flocculation phenotypes (Fig. 1). To address whether the flocculation is induced by silent-to-active conversions or transient derepression, we studied the locus using a yEGFP reporter and showed heterogenous yEGFP expression in the cells of each mutant strain (Fig. 2). Screening for yEGFP-positive flocs of cells also showed different floc sizes and differential yEGFP expression within the floc (Fig. 3A). However, the yEGFP signals in the mildly flocculating strains were significantly lower compared to those in the heavily flocculating *rrm3Δ asf1Δ* strain. These observations suggest that transient suppression of either the silent or active state, or both, is operating in these mutants. This conclusion would be further supported by the timelapse experiments in which, upon cell division, we observed the disappearance of yEGFP signal in *pol30-6 tof1Δ* (Fig. 3B). Furthermore, the observed effects in the single and double mutants are consistent with the notion that *pol30* mutations predispose cells to transient epigenetic instability, and only additional mutations in other replication factors lead to a complete conversion to an active state of the gene (Sauty and Yankulov, 2023).

In summary, our observations point to the possibility that the inheritance and reestablishment of heterochromatin at *FLO* loci is coupled with passage of the replication fork, and that the disruption in this process can cause a transient derepression or an epigenetic conversion.

### Mutations in *POL30* lead to transient derepression

Unlike the subtelomere, the mating-type and the rRNA loci, *FLO* loci experience *SIR*-independent silencing. Hence, studying the effects of *RRM3* and *TOF1* at *FLO11* in parallel with *VIIL* and *HMLα* allowed us to make conclusions about the nature of the effects independent of the silencing loci-specific trans-factors. A previous study using the CRASH assay has shown that the *pol30* mutations studied here cause transient loss of silencing at *HMLα* (Brothers and Rine, 2019). Here, we showed that a combination of *pol30* mutant alleles with *RRM3* or *TOF1* deletion causes extreme instability of the *HMLα* locus, thus rendering the CRASH assay too sensitive to address the nature of silencing defects in these mutants (Fig. 4). Instead, the analysis of silencing at the *VIIL* subtelomere using the *URA3-yEGFP* dual reporter showed transient effects (Fig. 5) (Sauty and Yankulov, 2023). The observations at *FLO11*, combined with the observations at *HMLα* and *VIIL* subtelomeres, point to the requirement of *POL30*–*TOF1* and *POL30*–*RRM3* functional interactions to maintain the silencing at both SIR-dependent and SIR-independent loci. Whether and how mutations in *POL30* affect the functions of the trans-acting factors at these loci remains to be addressed. Equally importantly, it is not known exactly how the *pol30* mutations studied here impede the functionality of the replication forks. We demonstrated that the *pol30-6 rrm3Δ* strain shows elevated silent-to-active conversions at *FLO11* but has little additive effect at the *VIIL* locus (Figs 2 and 5). Additionally, *pol30-8* has a significantly lower effect in the *rrm3Δ* and *tof1Δ* strains compared to the other *pol30* mutations. The genetic interactions reported here can be used as a starting point to finely dissect the role of *POL30* during elongation.

### Effects of the disruption of the local replication landscape on the expression of *FLO11*

Many of the mutations that cause derepression of *FLO11*, including the mutations in this study, encode components of the replication forks or associated histone chaperones (Rowlands et al., 2019; Shaban et al., 2023). Additionally, the deletions of *RRM3* and *TOF1* have been shown to have opposing effects on the stability of paused replication forks at the RFB at the rRNA loci (Baretić et al., 2020; Bastia et al., 2016; Ivessa et al., 2003). These considerations prompted us to test whether variations in the local replication landscape of *FLO11* would alter the expression of reporters inserted in this locus.

In a set of experiments, we showed that the proximity of *ARS1* leads to the reduction of yEGFP expression from the *FLO11* locus but does not alter the patterns of expression in the *pol30 tof1Δ* and *pol30 rrm3Δ* mutants (Fig. 6A). These outcomes probably reflect a multifaceted effect of the inserted *ARS1*. It is well established that the late-firing or inactive origins can act as proto-silencers and boost the repression of genes at the mating-type and at subtelomeric loci (Fourel et al., 2004; Rehman and Yankulov, 2009; Rusche et al., 2003). A gene repression effect of the inserted *ARS1* is certainly consistent with the reduced expression of yEGFP (compare Fig. 2D and Fig. 6A) but hampers the interpretation of whether replication opposite to the direction of transcription of *FLO11*, and, therefore, transcription-replication collisions, affect gene silencing. Indeed, we did not observe different patterns of expression in the mutants (Figs 2D and 6A), but this effect could be a consequence of the anti-silencing effects of the mutations studied in combination with pro-silencing effects of a weak or non-firing *ARS1* at this location. Further detailed analyses in other mutants and other loci are needed to definitively address this important question.

We also asked if the insertion of a RFB in the *FLO11* locus would alter the pattern of the expression of a reporter, in this case, a fusion RFB–RFP, in the *pol30* mutants harboring a deletion of *RRM3* or *TOF1* (Fig. 6B). We observed that the deletion of *TOF1*, but not *RRM3*, in the *pol30-6* background led to a significant derepression of *FLO11*. Although this outcome strongly suggests that the pausing of a replisome with a defective FPC (Tof1p–Csm3p–Mrc1p) affects gene silencing, the underlying mechanism remains enigmatic. The FPC aids the integrity of paused forks (Shyian et al., 2020). In addition, it has been recently shown that in mammals and *S. pombe*, the Mrc1p (claspin) subunit of FPC harbors histone chaperone activity and can be involved in the disassembly and reassembly of nucleosomes at the fork (Charlton et al., 2024; Yu et al., 2024). At this point, we can not address whether the fork pausing itself or an aberrant histone chaperone activity of FPC contributes to the observations in Fig. 6B. Another puzzling issue is the lack of effect of the deletion of *RRM3*. In earlier studies, we have shown that the deletion of *RRM3* alone has a weak effect on gene silencing at the subtelomeres (Wyse et al., 2016) and no effect at *FLO11* (Rowlands et al., 2019; Shaban et al., 2023) but has a strong effect at both loci upon the concomitant deletions of *ASF1* or *CAC1*. In these studies, we expressed the opinion that the effects of *RRM3* are not necessarily linked to its role in replication pausing, but to another yet unknown role in the replisome. The results in Fig. 6B support this earlier interpretation. Again, a detailed systematic approach is needed to address the connection between replication fork pausing, gene silencing and the role of *RRM3* in these processes.

### The role of lncRNA transcription in gene silencing

Loss of silencing by the passage of an impaired replication fork can be triggered by a combination of reasons, including disrupted recycling of parental histones, instability of the reassembled chromatin or the disruption of transcription of the regulating lncRNAs. Our results confirmed the previously reported link between the transcription of the *ICR1* and the silencing of *FLO11* (Bumgarner et al., 2012, 2009; Winston, 2009). At present, we cannot determine whether the passage of a defective replication fork or the reduced transcription of *ICR1* over the *FLO11* promoter has the primary effect on the silencing of *FLO11*. We also cannot exclude the possibility that the passage of a defective fork has an effect on the transcription of the lncRNAs upstream of the *FLO11* promoter. This latter possibility is quite exciting. Other lncRNAs, including *TERRA*, have been linked to the silencing of subtelomeric genes in yeasts and many other eukaryotes (Kwapisz and Morillon, 2020). It is possible that focused, in-depth studies at *FLO11* and the subtelomeric loci would reveal a link between the passage of the replication forks and the lncRNAs in the context of epigenetic regulation of gene expression. *S. cerevisiae* could again provide the stage for asking a fundamental question that so far has not been asked.

### Concluding remarks and significance

We show that mutations in the replicative clamp *POL30* (PCNA) predispose cells to epigenetic instability at *FLO* loci. We provide evidence that *RRM3* and *TOF1* genetically interact with *POL30* to maintain the chromatin structure of *FLO11*, as well as that of subtelomeres and mating-type loci. The effects of disturbing the local replication landscape on the silencing of *FLO11* could also be revealed in the analyzed mutants. We also show that mutations in *POL30*, *RRM3* and *TOF1* affect the abundance of the lncRNAs *ICR1* and *PWR1*. Our findings provide insights into the replication-coupled chromatin maintenance pathway and establishes *FLO11* as a beneficial locus to conduct further studies.

## MATERIALS AND METHODS

### Yeast strains

All *pol30* mutations are derived from the *W303* strain and obtained from Brothers and Rine (2019). *RRM3* and *TOF1* were disrupted in all strains using PCR-amplified disruption cassettes. Successful disruption of genes and insertions of reporters was confirmed by PCR. All strains were routinely maintained in YPD medium (1% yeast extract, 2% tryptone, 2% glucose) at 30°C. Strains for microscopy and flow cytometry were grown in synthetic complete (SC) medium to reduce the green fluorescence background. Strains with CRASH cassette were maintained in YPD supplemented with 200 μg/ml hygromycin B (GoldBio, H-270-1). All strains used in this study are listed in Table S1. All primers used to generate the knockout fragments are listed in Table S2.

### Reporter constructs

The *adh4-URA3-yEGFP←HTB1-tel* fragment was obtained as described in Sauty and Yankulov (2023); *URA3* is driven by its native promoter, whereas *yEGFP* is driven by the *HTB1* promoter. Strains with the CRASH construct were obtained from Brothers and Rine (2019).

Diagrams of constructs used for the modifications of the *FLO11* locus are shown in Fig. S4A. The *FLO11-yEGFP-KanMX* fragment was obtained as described previously (Rowlands et al., 2019; Shaban et al., 2023) and inserted at the *FLO11* locus, where the expression of *yEGFP* is driven by the native *FLO11* promoter. *FLO11-yEGFP-ARS1-URA3* was produced by amplifying the *ARS1-URA3* fragment from *pARS1* (Marahrens and Stillman, 1992) and replacing the *KanMX* gene in *FLO11-yEGFP-KanMX* with *ARS1-URA3*. *FLO11-RFB-RFP-HphMX* and *FLO11-BFR-RFP-HphMX* were produced by fusing in-frame upstream of the *RFP* ORF the sequence of two RFB sites derived from the rRNA replication pause site (Castán et al., 2017). The RFB sites were cloned in both orientations and preceded by 20 codons, thus positioning the first RFB 110 bases downstream of the *FLO11* TATA box. The RFBs can pause the fork in the *RFB* orientation and not in the *BFR* orientation (Castán et al., 2017). The sequences of the engineered *RFB-RFP* and *BFR-RFP* cassettes are shown in Fig. S4B.

### NAM assay

Cells were grown to saturation in 3 ml YPD cultures in the presence of 0, 2 and 5 mM NAM (Sigma-Aldrich, N0636). The sedimentation rate was calculated by resting the cells and recording the time required to clear the upper 50% of the culture volume (T^s50^) as recorded by visual observation. The sedimentation rates were expressed as T^s50^ at each NAM concentration divided by the T^s50^ of untreated cultures.

### Fluorescence microscopy

For suspension culture images, cells were grown in SC medium for 14 h. The cultures were vigorously shaken to disperse clusters and 2 µl of the suspension was examined on 8-well microscope slides. Images were captured by a Leica DM600B microscope equipped with a Hamamatsu Orca Flash 4.0 LT camera at 40× magnification. The Volocity software was used to measure GFP intensity and assess the number of GFP-positive cells. To determine the GFP signal intensity in individual cells, the pixel values of 100 cell-free regions of interest (ROIs) were averaged and considered as the background. Subsequently, the pixel values in ROIs of at least 100 isolated cells were measured and plotted in a distribution graph. The assessment of the percentage of GFP^+^ cells followed the method described in Shaban et al. (2023). Briefly, we measured the GFP intensities in cell-free ROIs, as well as cells with and without visible GFP signals. The intensities of the signals in cell-free ROIs were averaged and used to test arbitrary thresholds that can detect visibly positive and negative cells with >90% accuracy. Using these criteria, we postulated a threshold of 160% of average background intensity to detect GFP-positive cells. Cells exceeding this threshold were classified as GFP positive. Data from three independent experiments were pooled together to generate the distribution plot and perform percentage calculations.

For cell cluster images, cells were serially diluted in a glass-bottomed 96-well plate and grown without shaking for 24 h. The wells were imaged with an inverted Leica DMi8 microscope equipped with a 40× long working distance objective, 488 nm ILE laser and a monochrome Hamamatsu Orca Flash 4.0 camera. The images were processed with Volocity software.

### Timelapse microscopy

All strains were grown in YPD medium up to an optical density of 0.8–1 and serially diluted to obtain single cells. 2 μl of the diluted culture was spotted on SC agar medium. An agar slab was cut with a sterile spatula and placed inverted inside an 8-well microscopic chamber slide, which was then mounted on an inverted Nikon Eclipse Ti2 microscope equipped with a Hamamatsu Orca Flash 4.0 LT camera. A 40× long working distance objective was used with the Perfect Focus System (PFS) to image the cells every 10 min over 14 h to create timelapse movies. The temperature was maintained at 30°C with humidity using a Tokai Hit stage top incubator. The microscope, camera and stage were controlled by NIS Elements software version 5.30.04. All images were processed using NIS Elements AR version 5.21.03. Denoise.ai tool (within NIS Elements) was used to remove background noise. Brightfield to GFP signal ratios were adjusted for each strain to best visualize the GFP-positive cells. All movies were exported at a speed of 10 frames per second. Individual images were extracted using the ‘export image sequence’ tool in ImageJ 1.54d.

Epigenetic conversion was defined as the first appearance of a GFP-positive cell with a GFP intensity of 160% above the background. The intensity of the individual cells was quantified using the elliptical ROI tool of ImageJ. To calculate the number of divisions before epigenetic conversion, the number of cells was counted at the initial timepoint and at the timepoint of epigenetic conversion. The number of divisions was calculated using the following formula:

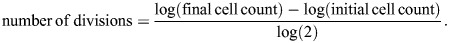


### CRASH assay

Cells harboring the CRASH reporter (Brothers and Rine, 2019) were grown in liquid culture containing 200 μg/ml hygromycin B to select for 100% RFP-positive cells. Cells were harvested, diluted in water, spread on a YPD plate and grown for 2–5 days. At least ten colonies were imaged by a Zeis Axiozoom.V16 microscope equipped with Hamamatsu Orca Flash 4.0 v3 cameras using a 1× objective. Images were captured and processed with Zen 2.6 Blue software.

### 5-FOA assay

Cells were cultured in YPD medium at 30°C overnight. Saturated cultures were diluted in a series of 1:10, and 5 μl of each dilution was spotted on YPD, synthetic complete medium without uracil (SC-Ura), 0.5× and 1×5-FOA plates with a final concentration of 5 mg/ml and 10 mg/ml 5-FOA (BioBasic, 703-95-7), respectively. All plates were then incubated at 30°C for 3–5 days, and colonies were counted using a Gallenkamp colony counter.

### Flow cytometry

Cells were cultured in SC medium until reaching an optical density at 600 nm (OD_600_) of 1; then, they were harvested and washed with phosphate-buffered saline. The resuspended cells underwent two cycles of sonication (30 s ON and 10 s OFF) using a Mandel Scientific ultrasonic sonicator at 50% output to break up cell clusters. GFP detection was carried out using a 488 nm laser on a Sony SH800z flow cytometer, and the LESH00SZFCPL software (Sony SH800z system software) was used to generate density plots and analyze the data. A screening of 100,000 events was conducted for each strain, with the GFP-negative gate established based on an isogenic strain lacking GFP. For experiments with the *FLO11-yEGFP-ARS1-URA3* construct, two replicates of flow cytometry were carried out using a 488 nm laser on a BD Accuri C6 flow cytometer, and the BD Accuri C6 software was used to analyze the data. A screening of approximately 300,000 events was conducted for these replicates, and the GFP-negative gate was established based on an isogenic strain lacking any fluorescent reporter construct.

### RT-qPCR

Cells were grown in YPD media to an OD_600_ of 1. 250 μl of packed harvested cells were resuspended in 750 μl of TRIzol (Thermo Fisher Scientific, 15596018) and crushed with 250 μl of 0.55 mm acid-washed glass beads (Cole-Parmer BioSpec, 11079105) at 4°C. 200 μl of chloroform was added to the lysates and they were spun for 15 min at 13,000 ***g***. The aqueous layer was collected and RNA was precipitated and extracted with 95% ethanol. The concentration and purity of RNA was determined using a NanoDrop 8000 (Thermo Fisher Scientific). First-strand cDNA synthesis was performed using SuperScript II Reverse Transcriptase (Thermo Fisher Scientific, 18064014) and the primers 5ʹ-CTCCACCACTGCTGAAAGAGAA-3ʹ for *ACT1*, 5ʹ-CCAGATTTGCCCAGCATTTC-3ʹ for *ICR1* and 5ʹ-CTTCCGCTCACAGGACAAA-3ʹ for *PWR1*. Subsequent quantitative PCR was performed using PowerUp SYBR Green Master Mix (Thermo Fisher Scientific, A25742) in an Applied Biosystems StepOnePlus thermocycler. Primers for qPCR are listed in Table S2. Primer efficiencies were between 95 and 110%. Each reaction was performed in triplicate for two biological replicates. The ΔΔCq method was used to quantify relative expression, where first the Cq values of *ICR1* and *PWR1* were normalized to the Cq value of *ACT1* within each strain (ΔCq). The ΔCq value for each strain and primer was then normalized to the Cq values of the wild-type *POL30-0* strain (ΔΔCq). Fold expression was calculated as 2^−ΔΔCq^ and plotted as a bar graph.

### Data analysis and statistics

All experiments were performed in triplicate unless otherwise specified. Calculations of mean and standard deviations were performed in RStudio version 4.3.1. Data analysis was performed using the *dplyr* package (doi:10.32614/CRAN.package.dplyr) and plotted using the *ggplot2* package (doi:10.32614/CRAN.package.ggplot2). One-way ANOVA followed by post hoc Dunnett's test was performed for multiple statistical comparisons using the DescTools package (doi:CRAN.package.DescTools). *P*-values <0.05 were considered as statistically significant.

## Supplementary Material

10.1242/joces.262006_sup1 Supplementary information
