# Supplementary material for: Mutations in the DNA processivity factor POL30 predispose the FLO11 locus to epigenetic instability in S. cerevisiae
Source: J Cell Sci. 2024 Dec 17;137(24):jcs262006. doi: 10.1242/jcs.262006 (PMC11827858; doi:10.1242/jcs.262006)
Supplement: Supplementary information [file joces-137-262006-s1.pdf]

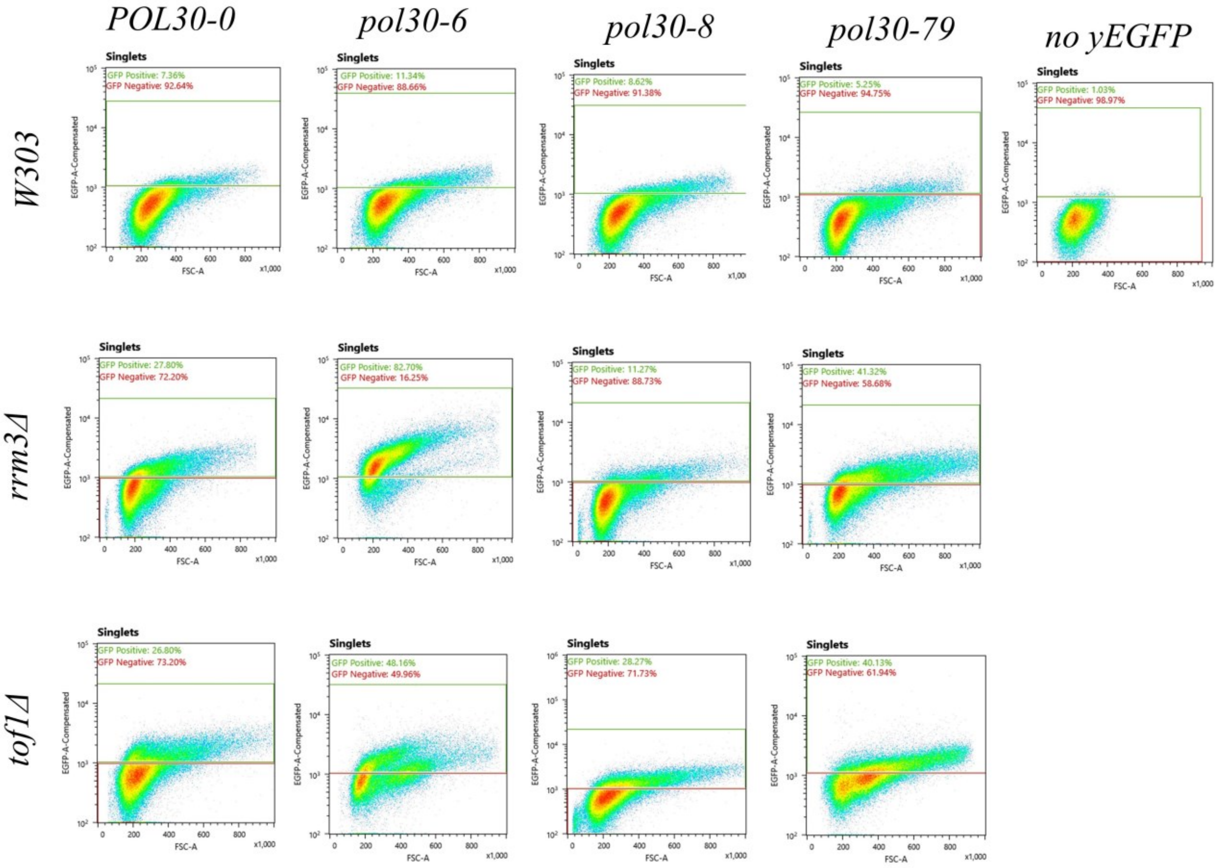

Fig. S1. Flow cytometry analysis of *FLO11-yEGFP* expression. Representative density plots of indicated strains are shown. The analysis of the raw data is presented in the bar graph in Fig. 2D.

Journal of Cell Science • Supplementary information

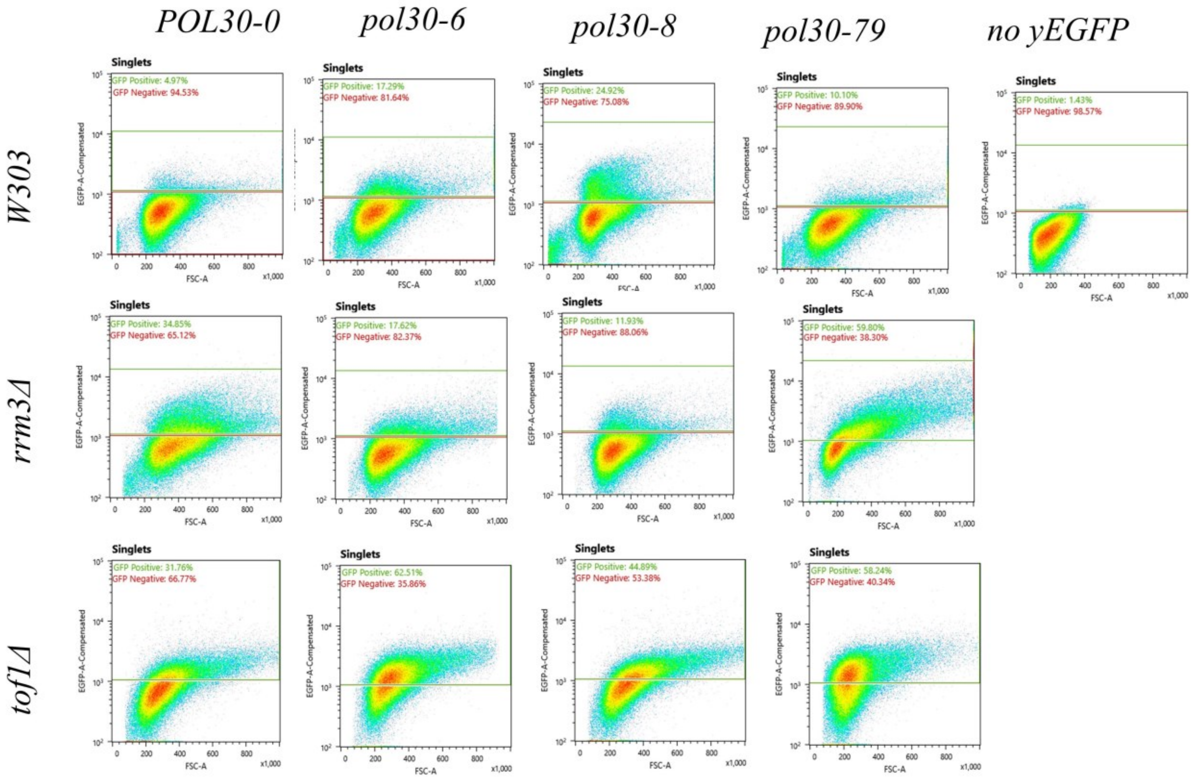

Fig. S2. Flow cytometry density plots using *adh4-URA3-yEGFP←HTB1-tel.* Representative density plots of indicated strains are shown. The analysis of the raw data is presented in the bar graph in Fig. 5B.

Journal of Cell Science • Supplementary information

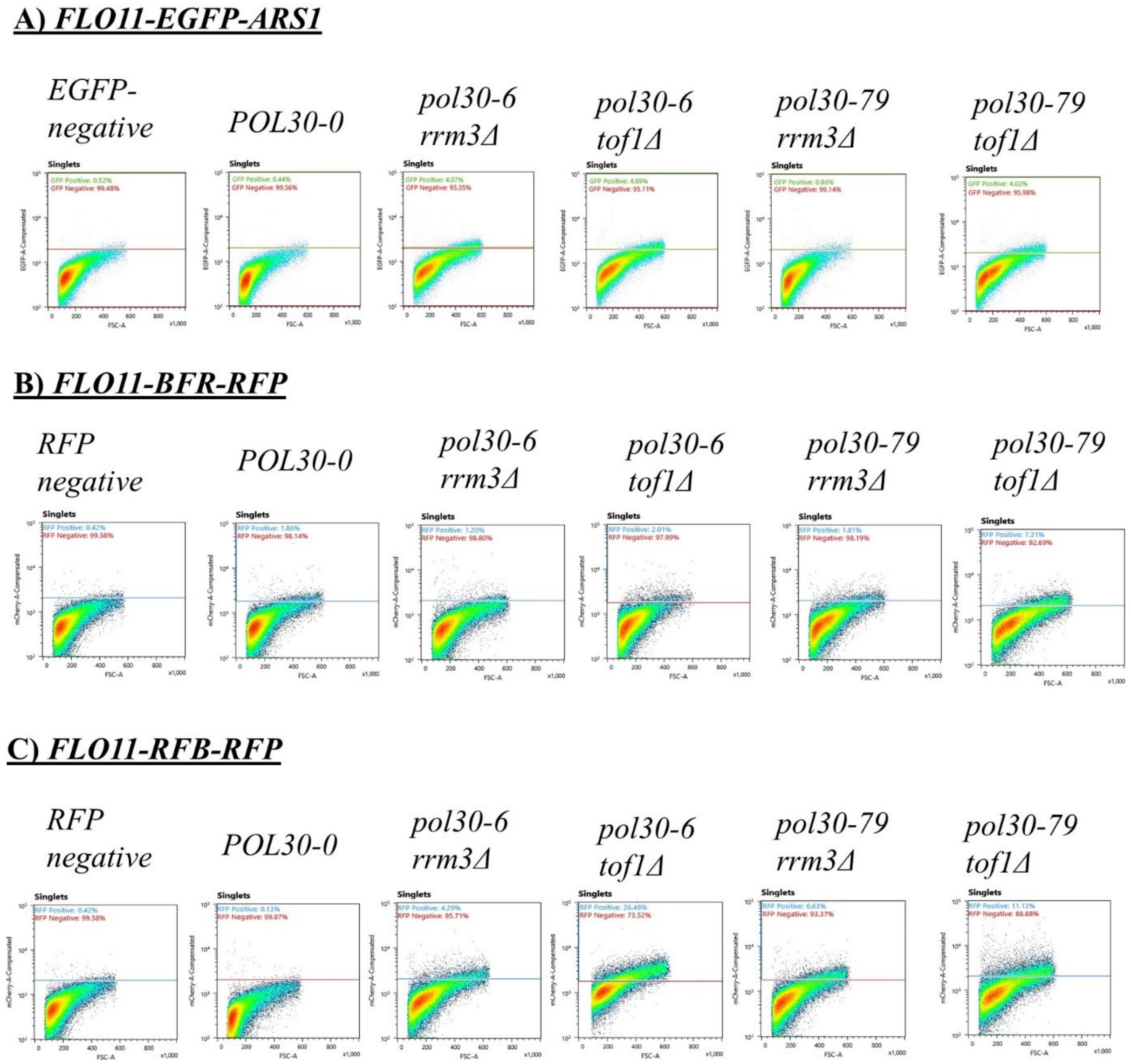

Fig. S3. Flow cytometry density plots using *FLO11-yEGFP-ARS1, FLO11-BFR-RFP* and *FLO11-RFB-RFP* reporters. Representative density plots of indicated strains are shown. The analysis of the raw data is presented in the bar graphs in Fig. 6.

Journal of Cell Science • Supplementary information

A)

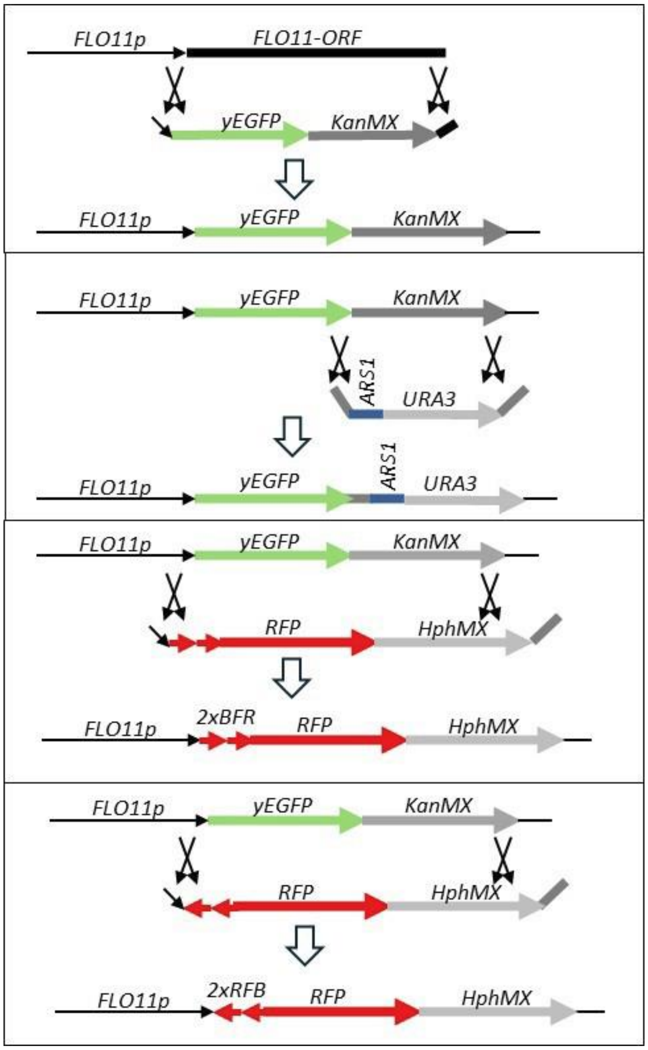

B) **RFB**:ATGCAGAAGCTGATCTCAGAGGAGGACCTGGGCCAGAAGCTGATCTCAGAGGA GGACCTG**AAACTTATACAAGCACTCATC**GGTGGCGGAGGGGGTGGCC**TTTGTGAA AGCCCTTCTCTC**ATGGTTTCAAAAGGTGAAGAAGATAATATGGCTATTATTAAAGAAT TTATGAGATTTAAAGTTCATATGGAAGG**...**

BFR:ATGCAGAAGCTGATCTCAGAGGAGGACCTGGGCCAGAAGCTGATCTCAGAGGA GGACCTG**ATGAGTGCTTGTATAAGTTT**GGGTGGCGGAGGGGGTGGC**AGAGAAGGG CTTTCACAAAGG**ATGGTTTCAAAAGGTGAAGAAGATAATATGGCTATTATTAAAGAAT TTATGAGATTTAAAGTTCATATGGAAGG**...**

Journal of Cell Science • Supplementary information

Fig. S4. Diagrams and sequence of the constructs for the modification of the *FLO11* locus.

A) Diagrams for the modification of the*FLO11* locus. *ARS1-URA3* fragment was derived from *pARS1* (Marahrens and Stillman, 1992). The two RFB sites are derived from (Castán et al., 2017).

B) Sequences of the 5' ends of the RFB-RFP and BFR-RFP reporter cassettes. The twoRFB (Replication Fork Barrier) sites that bind the Fob1p protein and are underlined. Thereplication fork is arrested only in the RFB orientation. The ORF of RFP (Red Fluorescent Protein) is shown in RED.

Journal of Cell Science • Supplementary information

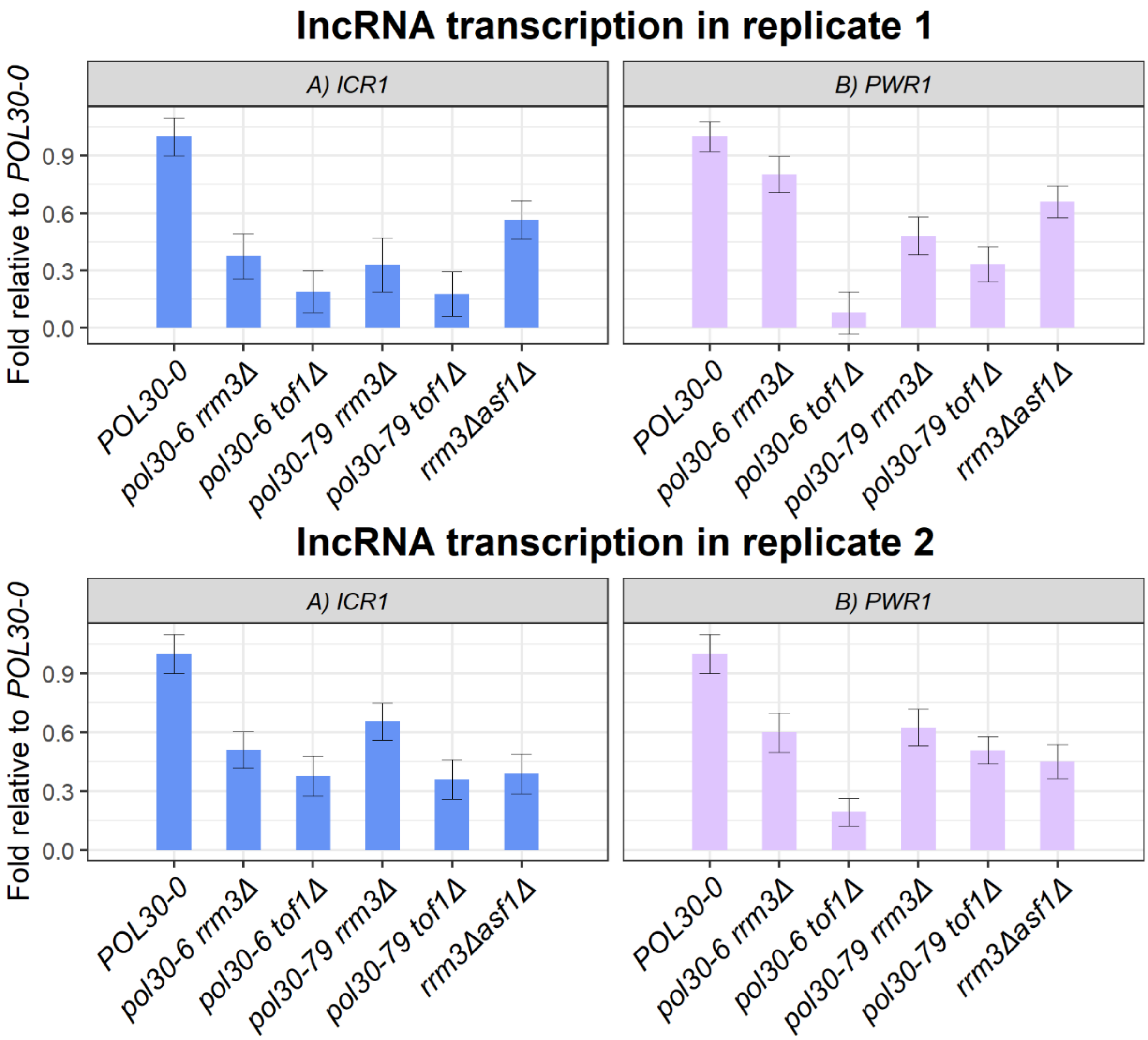

Fig. S5. Abundance of non-coding RNA in independent biological replicates. Error bars are representative of standard deviations in the technical replicates.

Journal of Cell Science • Supplementary information

Table S1. Strains used in this study

Strain	*MAT*	Genotype	Source
POL30-0	*a*	*can1-100, his3-11, leu2-3,112, trp1-1, ura3-1, lys2*	(Brothers & Rine, 2019)
pol30-6	*a*	*can1-100, his3-11,15, leu2-3,112, lys2, ura3-1, pol30-6*	(Brothers & Rine, 2019)
pol30-8	*a*	*can1-100, his3-11,15, leu2-3,112, lys2, ura3-1, pol30-8*	(Brothers & Rine, 2019)
pol30-79	*a*	*can1-100, his3-11,15, leu2-3,112, lys2, ura3-1, pol30-79*	(Brothers & Rine, 2019)
POL30-0 FLO11::yEGFP::KanMX	*a*	*POL30-0 FLO11::yEGFP::KanMX*	This study
pol30-6 FLO11::yEGFP::KanMX	*a*	*pol30-6 FLO11::yEGFP::KanMX*	This study
pol30-8 FLO11::yEGFP::KanMX	*a*	*pol30-8 FLO11::yEGFP::KanMX*	This study
pol30-79 FLO11::yEGFP::KanMX	*a*	*pol30-79 FLO11::yEGFP::KanMX*	This study
POL30-0 FLO11::yEGFP::KanMX tof1Δ::LYS2	*a*	*POL30-0 FLO11::yEGFP::KanMX tof1Δ::LYS2*	This study
pol30-6 FLO11::yEGFP::KanMX tof1Δ::Lys2	*a*	*pol30-6 FLO11::yEGFP::KanMX tof1Δ::LYS2*	This study
pol30-8 FLO11::yEGFP::KanMX tof1Δ::LYS2	*a*	*pol30-8 FLO11::yEGFP::KanMX tof1Δ::LYS2*	This study
pol30-79 FLO11::yEGFP::KanMX tof1Δ::LYS2	*a*	*pol30-79 FLO11::yEGFP::KanMX tof1Δ::LYS2*	This study
POL30-0 FLO11::yEGFP::KanMX rrm3Δ::HIS2	*a*	*POL30-0 FLO11::yEGFP::KanMX rrm3Δ::HIS2*	This study
pol30-6 FLO11::yEGFP::KanMX rrm3Δ::HIS2	*a*	*pol30-6 FLO11::yEGFP::KanMX rrm3Δ::HIS2*	This study
pol30-8 FLO11::yEGFP::KanMX rrm3Δ::HIS2	*a*	*pol30-8 FLO11::yEGFP::KanMX rrm3Δ::HIS2*	This study
pol30-79 FLO11::yEGFP::KanMX rrm3Δ::HIS2	*a*	*pol30-79 FLO11::yEGFP::KanMX rrm3Δ::HIS2*	This study
BY4742 FLO11::yEGFP::KanMX rrm3Δ::LEU2 asf1Δ::HIS3	α	*leu2Δ ura3Δ his3Δ lys2Δ FLO11::yEGFP::KanMX rrm3Δ::LEU2 asf1Δ::HIS3*	(Shaban *et al.*, 2023)

Journal of Cell Science • Supplementary information

POL30-0 adh4-URA3-yEGFP←HTB1 VIIL-tel	*a*	*POL30-0 adh4-URA3-yEGFP←HTB1 VIIL-tel*	(Sauty &Yankulov, 2023)
pol30-6 adh4-URA3-yEGFP←HTB1 VIIL-tel	*a*	*pol30-6 adh4-URA3-yEGFP←HTB1 VIIL-tel*	(Sauty &Yankulov, 2023)
pol30-8 adh4-URA3-yEGFP←HTB1 VIIL-tel	*a*	*pol30-8 adh4-URA3-yEGFP←HTB1 VIIL-tel*	(Sauty &Yankulov, 2023)
pol30-79 adh4-URA3-yEGFP←HTB1 VIIL-tel	*a*	*pol3079- adh4-URA3-yEGFP←HTB1 VIIL-tel*	(Sauty &Yankulov, 2023)
POL30-0 rrm3Δ::KanMX adh4-URA3-yEGFP←HTB1 VIIL-tel	*a*	*POL30-0 rrm3Δ::KanMX adh4-URA3-yEGFP←HTB1 VIIL-tel*	(Sauty &Yankulov, 2023)
pol30-6 rrm3Δ::KanMX adh4-URA3-yEGFP←HTB1 VIIL-tel	*a*	*pol30-6 rrm3Δ::KanMX adh4-URA3-yEGFP←HTB1 VIIL-tel*	(Sauty &Yankulov, 2023)
pol30-8 rrm3Δ::KanMX adh4-URA3-yEGFP←HTB1 VIIL-tel	*a*	*pol30-8 rrm3Δ::KanMX adh4-URA3-yEGFP←HTB1 VIIL-tel*	(Sauty &Yankulov, 2023)
pol30-79 rrm3Δ::KanMX adh4-URA3-yEGFP←HTB1 VIIL-tel	*a*	*pol30-79 rrm3Δ::KanMX adh4-URA3-yEGFP←HTB1 VIIL-tel*	(Sauty &Yankulov, 2023)
POL30-0 tof1Δ::KanMX adh4-URA3-yEGFP←HTB1 VIIL-tel	*a*	*POL30-0 tof1Δ::KanMX adh4-URA3-yEGFP←HTB1 VIIL-tel*	This study
pol30-6 tof1Δ::KanMX adh4-URA3-yEGFP←HTB1 VIIL-tel	*a*	*pol30-6 tof1Δ::KanMX adh4-URA3-yEGFP←HTB1 VIIL-tel*	This study
pol30-8 tof1Δ::KanMX adh4-URA3-yEGFP←HTB1 VIIL-tel	*a*	*pol30-8 tof1Δ::KanMX adh4-URA3-yEGFP←HTB1 VIIL-tel*	This study
pol30-79 tof1Δ::KanMX adh4-URA3-yEGFP←HTB1 VIIL-tel	*a*	*pol30-79 tof1Δ::KanMX adh4-URA3-yEGFP←HTB1 VIIL-tel*	This study
POL30-0 CRASH	*a*	*POL30-0 hmlα2Δ::cre, ura3Δ::RFP-GFP(HphMX)*	(Brothers & Rine, 2019)
pol30-6 CRASH	*a*	*pol30-6 hmlα2Δ::cre, ura3Δ::RFP-GFP(HphMX)*	(Brothers & Rine, 2019)
pol30-8 CRASH	*a*	*pol30-8 hmlα2Δ::cre, ura3Δ::RFP-GFP(HphMX)*	(Brothers & Rine, 2019)
pol30-79 CRASH	*a*	*pol30-79 hmlα2Δ::cre, ura3Δ::RFP-GFP(HphMX)*	(Brothers & Rine, 2019)
POL30-0 rrm3Δ::KanMX CRASH	*a*	*POL30-0 rrm3Δ::KanMX hmlα2Δ::cre, ura3Δ::RFP-GFP(HphMX)*	This study
pol30-6 rrm3Δ::KanMX CRASH	*a*	*pol30-6 rrm3Δ::KanMX hmlα2Δ::cre, ura3Δ::RFP-GFP(HphMX)*	This study

Journal of Cell Science • Supplementary information

pol30-8 rrm3Δ::KanMX CRASH	*a*	*pol30-8 rrm3Δ::KanMX hmlα2Δ::cre, ura3Δ::RFP-GFP(HphMX)*	This study
pol30-79 rrm3Δ::KanMX CRASH	*a*	*pol30-79 rrm3Δ::KanMX hmlα2Δ::cre, ura3Δ::RFP-GFP(HphMX)*	This study
POL30-0 tof1Δ::KanMX CRASH	*a*	*POL30-0 tof1Δ::KanMX hmlα2Δ::cre, ura3Δ::RFP-GFP(HphMX)*	This study
pol30-6 tof1Δ::KanMX CRASH	*a*	*pol30-6 tof1Δ::KanMX hmlα2Δ::cre, ura3Δ::RFP-GFP(HphMX)*	This study
pol30-8 tof1Δ::KanMX CRASH	*a*	*pol30-8 tof1Δ::KanMX hmlα2Δ::cre, ura3Δ::RFP-GFP(HphMX)*	This study
pol30-79 tof1Δ::KanMX CRASH	*a*	*pol30-79 tof1Δ::KanMX hmlα2Δ::cre, ura3Δ::RFP-GFP(HphMX)*	This study
POL30-0 FLO11-BFR-RFP-HphMX	*a*	*POL30-0 FLO11::BFR-RFP- HphMX*	This study
POL30-0 FLO11-RFB-RFP HphMX	*a*	*POL30-0 FLO11::RFB-RFP-HphMX*	This study
pol30-6 FLO11-BFR-RFP-HphMX tof1Δ	*a*	*pol30-6 FLO11::BFR-RFP-HphMX tof1Δ::LYS2*	This study
pol30-6 FLO11-RFB-RFP-HphM tof1Δ	*a*	*pol30-6 FLO11::RFB-RFP- HphMX tof1Δ::LYS2*	This study
pol30-6 FLO11-BFR-RFP-HphMX rrm3Δ	*a*	*pol30-6 FLO11::BFR-RFP- HphMX rrm3Δ::HIS2*	This study
pol30-6 FLO11-RFB-RFP HphMX rrm3Δ	*a*	*pol30-6 FLO11::RFB-RFP-HphMX rrm3Δ::HIS2*	This study
pol30-79 FLO11-BFR-RFP-HphMX tof1Δ	*a*	*pol30-79 FLO11::BFR-RFP-HphMX tof1Δ::LYS2*	This study
pol30-79 FLO11-RFB-RFP HphM tof1Δ	*a*	*pol30-79 FLO11::RFB-RFP-HphMX tof1Δ::LYS2*	This study
pol30-79 FLO11-BFR-RFP-HphMX rrm3Δ	*a*	*pol30-79 FLO11::BFR-RFP-HphMX rrm3Δ::HIS2*	This study
pol30-79 FLO11-RFB-RFP-HphM rrm3Δ	*a*	*pol30-79 FLO11::RFB-RFP-HphMX rrm3Δ::HIS2*	This study
POL30-0 FLO11-yEGFP-ARS1	*a*	*POL30-0 FLO11::yEGFP-ARS1-URA3*	This study
pol30-6 FLO11-yEGFP-ARS1 tof1Δ	*a*	*pol30-6 FLO11:y:EGFP-ARS1-URA3 tof1::LYS2*	This study
pol30-6 FLO11-yEGFP-ARS1 rrm3Δ	*a*	*pol30-6 FLO11::yEGFP-ARS1-URA3 rrm3::HIS2*	This study
pol30-79 FLO11-EGFP-ARS1 tof1Δ	*a*	*pol30-79 FLO11::yEGFP-ARS1-URA3 tof1::LYS2*	This study
pol30-79 FLO11-yEGFP-ARS1 rrm3Δ	*a*	*pol30-79 FLO11::yEGFP-ARS1-URA3 rrm3::HIS2*	This study

Journal of Cell Science • Supplementary information

Table S2. Primers used in this study.

Primer	Sequence	Use
FLO11 F	GCCTTACAAAATTATGGCAGCG	*FLO11::yEGFP::KanMX* fragment generation
FLO11 R	TGTTGAAGGGTTCCCAATTGA	*FLO11::yEGFP::KanMX* fragment generation
RRM3 F	GCCAACATTTCGCAGTCTTC	Knockout fragment generation
RRM3 R	ATAACGGGGCTAACCCGAAT	Knockout fragment generation
TOF1 F	GGAAATGGCCCTTCCATTAT	Knockout fragment generation
TOF1 R	TGATAAAGAATGAATCGCATGT	Knockout fragment generation
ICR1 F	CCAGATTTGCCCAGCATTTC	RT-qPCR
ICR1 R	GGTGGTGAGAACCATCACTAAT	RT-qPCR
PWR1 F	CTTCCGCTCACAGGACAAA	RT-qPCR
PWR1 R	GAAGTGCAGTGAGAGGAAGAG	RT-qPCR
ACT1 F	CTCCACCACTGCTGAAAGAGAA	RT-qPCR
ACT1 R	CCAAGGCGACGTAACATAGTTTT	RT-qPCR
RFP-5FWD	ATGGTTTCAAAAGGTGAAGAAGATA ATATGGC	Forward primer for knockout fragment generation from *pFOM298* plasmid containing *RFP-HphMX*
HphMX-KanMX disr	GCGCCTGAGCGAGACGAAATACGC GATCGCTGTTAAAAGGACAATTACA AACAGGAATCGAATGCAACCGGCG CACTTAACTTCGCATCTGGG	Reverse primer for knockout generation from *pFOM298* plasmid containing *RFP-HphMX*
KanMX-REV	GCGCCTGAGCGAGACGAAATAC	Reverse primer for amplifying the *RFP-HphMX* assembled fragment for sequencing
FLO11p-5-FWD	CGTATAAAAAGCACCCTATTCATCA GT	Forward primer for amplifying the *RFP-HphMX* assembled fragment for sequencing
BFR-RFP-HphMX	ATGAGTGCTTGTATAAGTTTGGGTG GCGGAGGGGGTGGCAGAGAAGGGC TTTCACAAAGGGTTTCAAAAGGTGA AGAAGATAATATGGC	primer for inserting *BFR* into 5' end of the synthetic fragment
RFB-RFP-HphMX	ATGAAACTTATACAAGCACTCATCG GTGGCGGAGGGGGTGGCCTTTGTGA AAGCCCTTCTCTCGTTTCAAAAGGT GAAGAAGATAATATGGC	primer for inserting *RFB* into 5' end of the synthetic fragment
RFB-RFP-HphMX-FLO11	ATCCCTCGTCATGTTGTGGTTCTAAT TAAAATATACTTTTGTAGGCCTCAA AAATCCATATACGCACACTATGAGT GCTTGTATAAGTTTGGGTGG	Adds 5' *FLO11* homologous region to *RFB-RFP* synthetic fragment

Journal of Cell Science • Supplementary information

BFR-RFP-HphMX-FLO11	ATCCCTCGTCATGTTGTGGTTCTAAT TAAAATATACTTTTGTAGGCCTCAA AAATCCATATACGCACACTATGAAA CTTATACAAGCACTCATCGGTGG	Adds 5’ *FLO11* homologous region to *BFR-RFP* synthetic fragment
5’ URA3-KanMX disr	CCATACATCCCCATGTATAATCATTT GCATCCATACATTTTGATGGCCGCA CGGCGCGAAGCAAAAATTAGTTGTA AAACGACGGCCAGTG	Forward primer for amplifying *ARS1* fragment from *pARSwt* plasmid and adding *URA3* into the fragment
3’ M13-ARS1-URA3	GCGCCTGAGCGAGACGAAATACGC GATCGCTGTTAAAAGGACAATTACA AACAGGAATCGAATGCAACCGGCG CGCGTTGGCCGATTCA	Reverse primer for amplifying *ARS1* fragment from *pARSwt* plasmid

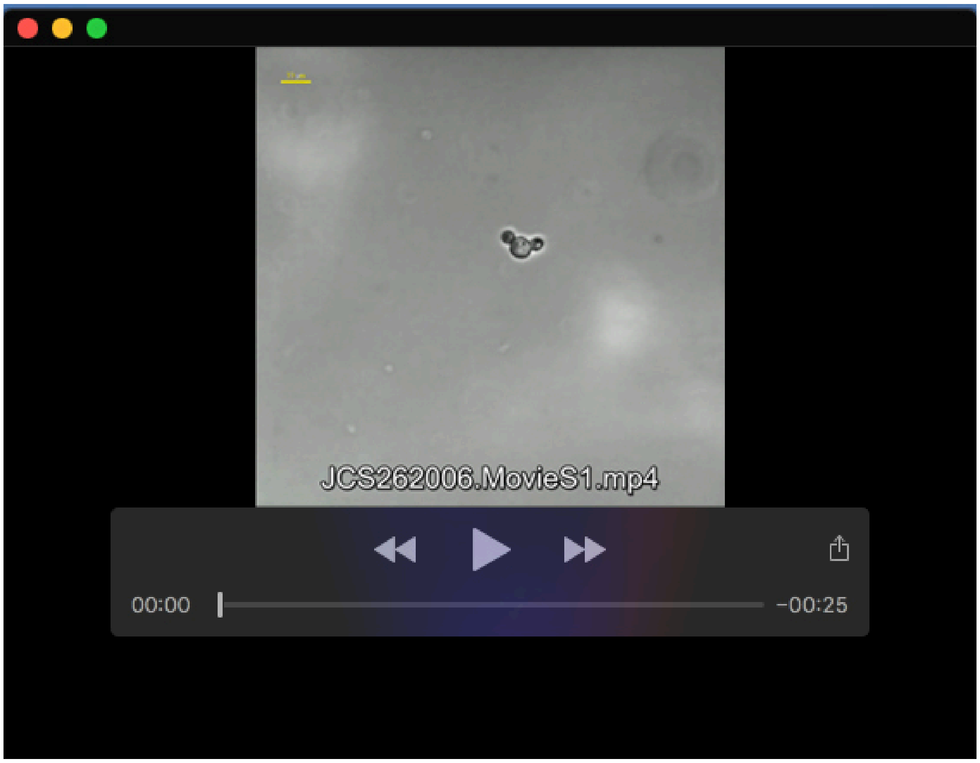

Movie 1. Time lapse movie of *a pol30-6 rrm3Δ* cell harboring *FLO11-yEGFP* imaged over 14 hours. Frame rate=10 FPS. Scale bar=10 μm.

Journal of Cell Science • Supplementary information
